# Systematic analysis of the role and significance of target genes of active ingredients of traditional Chinese medicine injections in the progression and immune microenvironment of hepatocellular carcinoma

**DOI:** 10.3389/fphar.2022.1095965

**Published:** 2023-01-06

**Authors:** Chao Wang, Lili Yang, Shaoheng Xu, Hui Guo, Hewen Guan, Qiannan Wang, Xueyan Jiang, Mingyang Fei, Jinbao Zhang

**Affiliations:** ^1^ Department of General Surgery, First Affiliated Hospital of Dalian Medical University, Dalian, Liaoning, China; ^2^ Department of Dermatology, First Affiliated Hospital of Dalian Medical University, Dalian, Liaoning, China; ^3^ Department of Plastic Surgery, First Affiliated Hospital of Dalian Medical University, Dalian, Liaoning, China; ^4^ Department of Neurosurgery, First Affiliated Hospital of Dalian Medical University, Dalian, Liaoning, China

**Keywords:** hepatocellular carcinoma, traditional Chinese medicine, immune microenvironment, injection, adjuvant therapy

## Abstract

**Background:** Traditional Chinese medicine in China is an important adjuvant therapy for the treatment of hepatocellular carcinoma (HCC) and traditional Chinese medicines injections have a wide range of clinical applications. The purpose of this study was to identify the active ingredients and related genes of traditional Chinese medicine injections that can treat hepatocellular carcinoma.

**Methods:** Effective small molecule components were extracted from 14 types of traditional Chinese medicines from 8 injections and the main gene targets were identified. The 968 patients with HCC were classified based on the target gene set, and the characteristics of patients with different subtypes were analyzed. Patients with two subtypes of HCC were compared with normal tissues and cirrhosis to identify important gene targets related to traditional Chinese medicines in HCC progression.

**Results:** In this study, 138 important genes associated with traditional Chinese medicines were identified and two HCC subtypes were identified. By analyzing the differences between the two subtypes, 25 related genes were associated with HCC subtypes. Through clinical and pharmacological analysis, this study identified quercetin as an important traditional Chinese medicines small molecule and secreted phosphoprotein 1 (SPP1) as an important oncogene in HCC.

**Conclusion:** Traditional Chinese medicines injection is an important adjuvant treatment modality for HCC. SPP1 is an important oncogene in HCC.

## Introduction

Liver cancer is the sixth leading cause of death in the world, accounting for about 8.3% of cancer mortality (Sung et al., 2021). Liver cancer is a common chronic liver disease. Hepatitis viruses HBV and HCV are common causes of liver cancer. Liver cirrhosis caused by virus can develop into liver cancer under the action of various incentives. Liver cancer caused by hepatitis virus is also a common type of liver cancer in the Chinese population (Shi et al., 2005; Schietroma et al., 2018). At the same time, excessive intake of aflatoxin, alcoholic liver disease and non-alcoholic fatty liver disease are also common causes of liver cancer (Sagnelli et al., 2020; Llovet et al., 2021).Most of these diseases can cause liver fibrosis and chronic inflammation, thereby promoting the occurrence and development of liver cancer (Dhar et al., 2020). Hepatocellular carcinoma (HCC) is a liver tumor originating from hepatocytes and is the most common type of liver cancer. 90% of liver cancers are hepatocellular carcinoma (Gao et al., 2020). Traditional treatments for HCC include surgery, chemotherapy, radiation therapy, targeted therapy, etc., (Wang et al., 2019). In recent years, with the emergence of immunotherapy, many immunotherapy drugs, such as PD-L1/PD-1 monoclonal antibody, have been widely used in the treatment of liver cancer. At present, immunotherapy combined with traditional targeted therapy has become an important treatment method for HCC (Makarova-Rusher et al., 2015; De Martin et al., 2018; Xu et al., 2018; Faivre et al., 2020).

Traditional Chinese medicine is a traditional Chinese medicine treatment method. Many studies have shown that traditional Chinese medicine has a greater role in a variety of tumor microenvironments. Many active ingredients of traditional Chinese medicine can promote the generation of tumor immune cells and the occurrence of anti-tumor responses (Zhai et al., 2019; Wang et al., 2020a). Traditional Chinese medicine has a variety of anti-tumor effects in the treatment of HCC and can play a greater role in other therapeutic methods (Wei et al., 2022). However, traditional Chinese medicines are often administered orally or externally due to the complex composition of traditional Chinese medicines. In this way, the effect of some traditional Chinese medicines is not obvious and it is difficult to study the main active components of traditional Chinese medicines. In recent years, with the discovery of traditional Chinese medicine ingredients and the improvement of refining technology, more traditional Chinese medicine injections have appeared in clinical treatment. In this study, 8 kinds of commonly used and commercialized traditional Chinese medicine injections were collected, and these injections were all related to hepatocellular carcinoma. Ideas and results.

## Method and material

### Classification of traditional Chinese medicine and acquisition of active ingredients

In this study, the commonly used traditional Chinese medicine injections were screened, and 8 traditional Chinese medicine injections related to HCC were obtained. Eight kinds of traditional Chinese medicine injections contain a total of 14 kinds of traditional Chinese medicines. According to the medicinal properties and pharmacological effects of traditional Chinese medicines, we divided the 14 kinds of traditional Chinese medicines into three categories. The first category is heat-clearing traditional Chinese medicine (HCM), including marsdenia tenacissima, sarcandra glabra, semen coicis, venenum bufonis and mylabris. The second category is tonic traditional Chinese medicine (TCM), including ginseng, astragalus mongholicus, red ginseng and acanthopanax root. The third category of pain-relieving traditional Chinese medicine (PRCM), including caulis sinomenii, celandine, schefflera kwangsiensis, aconiti radix and radix aconiti agrestis.

We obtained the main components of relevant Chinese medicines from 4 Chinese medicine databases including SymMap, TCMID, TCMSP, and TCM-ID. The pharmacokinetics and pharmacokinetics of these components were obtained from the TCMSP database. We screened the main components with DL≧0.1 and TPSA≤140 as the active ingredients of various traditional Chinese medicines. Among the three types of traditional Chinese medicines, 50 active ingredients were screened by HCM; 153 active ingredients were screened by TCM; 110 active ingredients were screened by PRCM.

### Acquisition of related genes in active ingredients of traditional Chinese medicine

In this study, the target of 303 main components of three types of traditional Chinese medicines was determined through the four traditional Chinese medicine databases of SymMap, TCMID, TCMSP and TCM-ID. Then, we obtained the HCC-related target sets through the DisGNET database and the GeneCard database, and obtained the intersections with the three types of traditional Chinese medicines targets respectively. The three target gene sets were intersected and 138 target genes were found.

### RNA-seq data of HCC patients and correlation analysis

In this study, RNA-seq data were collected from three online databases, TCGA(TCGA-LIHC), ICGC(ICGC-LIRI) and GEO (GSE14520,GSE116174,GSE54236), for 968 cases of patients with HCC. RNA-seq data from another 340 patients with cirrhosis were also collected for comparison in GEO (GSE15654,GSE84044). A total of 479 cases of RNA-seq data from normal liver tissues were obtained from three online databases, TCGA(TCGA-LIHC), GTEx and GEO (GSE14520,GSE54236). All sequencing data were transformed into FPKM data and normalized accordingly. Spearman test was used for all gene expression correlation analysis in this study. In this study, the expression of 120 target genes in HCC was demonstrated.

### Consistent clustering and variance analysis

Based on the expression of 120 target genes, 968 patients were divided into Class I and Class II by consistent clustering method. Then, the differential genes between the two groups were detected by DeSeq2 differential gene analysis. Differential gene screening conditions were *p* < 0.05 and log2FC < -1 or log2FC > 1. WGCNA analysis was used to further identify important sets of differential genes between the two HCC subtypes.

### Gene and pathway enrichment analysis

GSEA enrichment analysis was performed on the differential genes of the two types of samples, and gene functions were annotated by using the gene sets of GO and KEGG. At the same time, the basic enrichment analysis was performed on the target gene sets of three types of traditional Chinese medicines by using the gene sets of GO and KEGG. GSVA pathway analysis was used to analyze the differences in pathway enrichment between the two HCC subtypes.

### Immune infiltration analysis and survival analysis

The Cibersort immune infiltration analysis was performed on the gene sets of the two types of samples, and the differences in the infiltration degrees of 22 immune cells in the two types of samples were obtained. 576 patients with HCC had clinical survival data. KM analysis was used to analyze the effect of HCC subtypes on survival of patients. Cox analysis was used to identify important genes associated with traditional Chinese medicines target genes that are associated with survival of patients with HCC.

### Genetic multi-omics analysis and drug analysis

The GEPIA database is used to observe gene expression in a variety of cancers. The ULCAN database is used to analyze gene methylation. cBioPortal database is used to analyze gene mutations. HPA data is used to analyze gene expression and cellular localization at the protein level. Oncopredict package and easier package are used to analyze different subtypes of HCC patients’ sensitivity to common small molecule drug treatments and immunotherapy. A molecular docking approach was used to validate the interactions between important small molecule components of traditional Chinese medicines and gene target proteins.

### Statistical analysis

The software used in this study includes R studio (R4.2.1), Cytoscape 3.7.2, Prism 9, Autodock4, Pymol and SPSS 26. Statistical results at *p* < 0.05 were considered statistically significant.

## Result

### Classification and target screening of traditional Chinese medicines related to HCC

**FIGURE 1:**
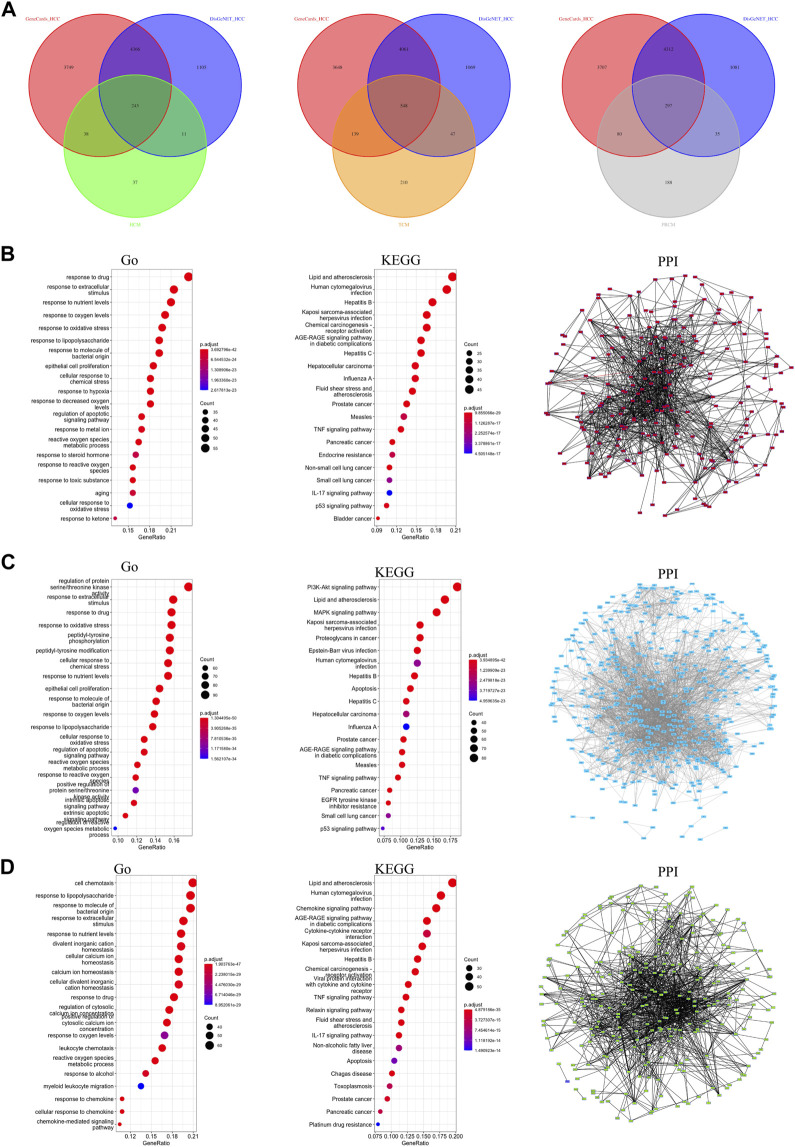
Classification and target screening of traditional Chinese medicines. **(A)** The number of HCC-related targets of three types. **(B)** Gene function analysis of HCM gene targets. **(C)** Gene function analysis of TCM gene targets. **(D)** Gene function analysis of PRCM gene targets.

In this study, we included 8 clinically used traditional Chinese medicine injections and collected small molecules and effective targets of 14 classes of Chinese medicines (Supplementary Table S1). A detailed analysis flow chart has been shown in Supplementary Figure S1. We divided the 14 classes of traditional Chinese medicine into three major categories based on the pharmacology and clinical efficacy of Chinese medicine. The first group of traditional Chinese medicine is HCM, which has the effect of clearing heat and detoxifying the body, and can inhibit the development of inflammation and cancer. The number of HCC-related targets of HCM is 243 (Figure 1A). The second group of traditional Chinese medicine is TCM, which has the effect of benefiting the immune function of the body and promoting the body’s anti-disease function. The number of HCC-related targets of TCM is 548 (Figure 1A). The third group of traditional Chinese medicine is PRCM, which has pain-relieving effect and is often used in the treatment of cancer pain in advanced cancer. The number of HCC-related targets of PRCM is 297 (Figure 1A). Gene enrichment analysis showed that the targets of HCM were related to drug response (Figure 1B); the targets of TCM were related to extracellular stimulation (Figure 1C); and the targets of PRCM were related to cell chemotaxis (Figure 1D). All three types of TCM were associated with hepatitis virus (Figures 1B–D). PPI analysis showed that the target sets of all three types of traditional Chinese medicine had high correlation (Figures 1B–D).

### Common targets of traditional Chinese medicine and classification subtypes of HCC

**FIGURE 2:**
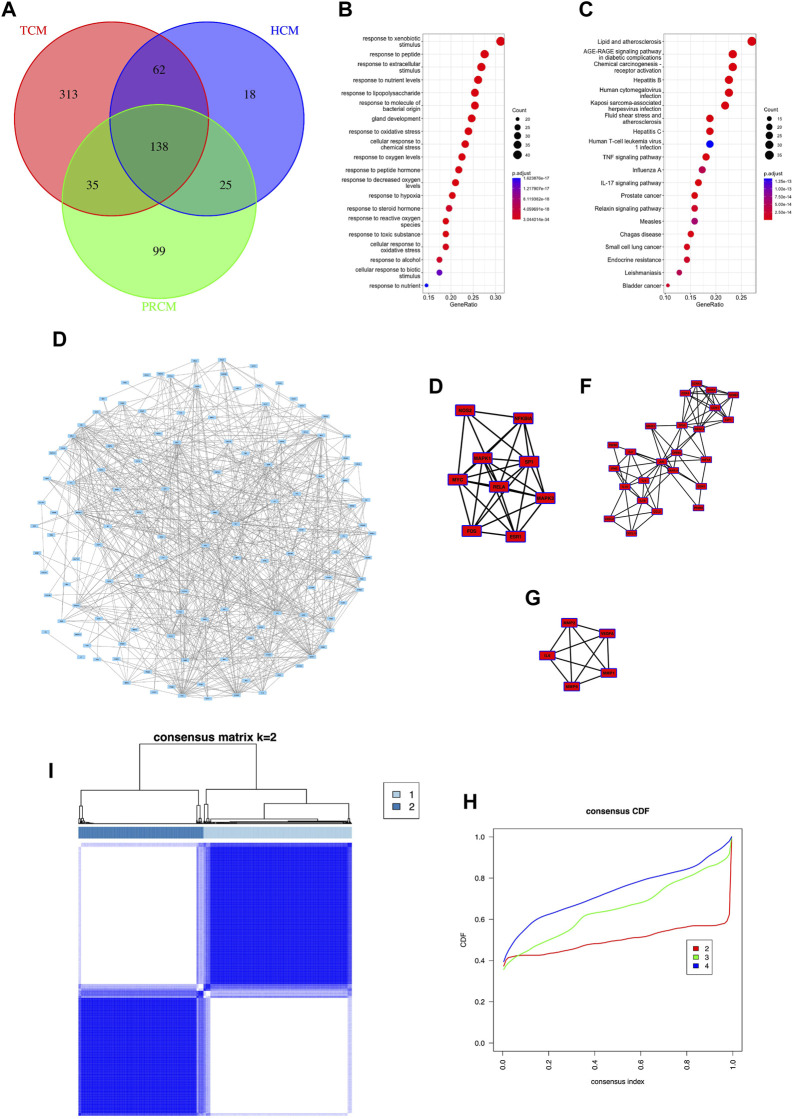
Common targets and classification subtypes. **(A)** The number of common targets. **(B)** Gene enrichment Go analysis of common targets. **(C)** Gene enrichment KEGG analysis of common targets. **(D)** PPI analysis of common targets. **(E)** Core networks 1 of PPI analysis. **(F)** Core networks 2 of PPI analysis.**(G)** Core networks 3 of PPI analysis. **(G)** Consensus matrix of consistent clustering. **(H)** Consensus CDF of consistent clustering.

In this study, 138 gene targets were obtained from the target sets of three types of traditional Chinese medicine (Figure 2A). Based on 138 genes, we collected related traditional Chinese medicine and small molecule components (Supplementary Table S2).Gene enrichment analysis showed that most of the 138 targets were associated with cellular stress response, hepatitis virus (Figures 2B,C). PPI analysis showed a large correlation between targets (Figure 2D) and three core networks were obtained with clustering indices of 7.5 (Figure 2E), 6.87 (Figure 2F), and 5 (Figure 2G), respectively. Most of the genes in all three core networks were associated with cellular stress response and cellular inflammation. In this study, sequencing data from 968 HCC cases were used to find the expression of 120 targets (The details are shown in Supplementary Table S3). Therefore, based on the expression of 120 targets, consistent clustering was performed and patients with HCC were classified into two subtypes, Class I and Class II (Figures 2H,I).

### Target expression and differential analysis of different subtypes of HCC

**FIGURE 3:**
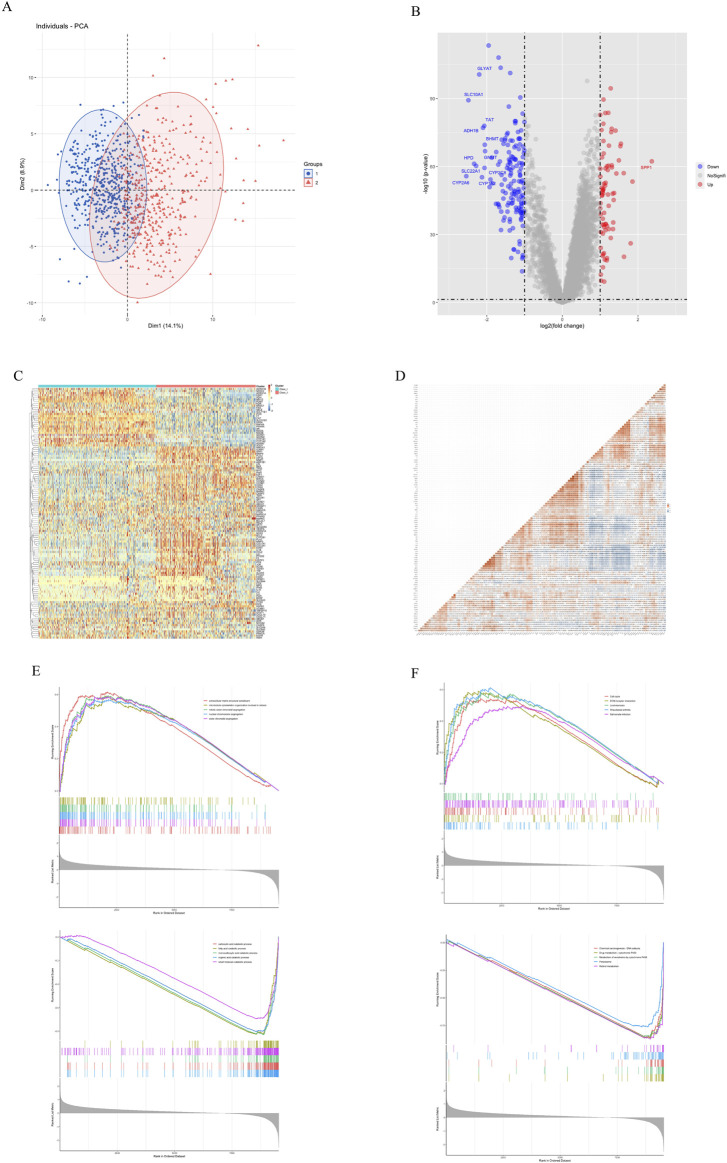
Gene differential analysis of two subtypes. **(A)** PCA analysis. **(B)** Volcano map of differential analysis. **(C)** Expression of 120 traditional Chinese medicine targets. **(D)** Correlation of 120 traditional Chinese medicine targets. **(E)** GSEA Go analysis. **(F)** GSEA KEGG analysis.

PCA analysis showed that based on the expression of traditional Chinese medicine targets, 968 patients could be clearly classified into two categories, with 525 cases in Class I and 443 cases in Class II (Figure 3A). Differential analysis showed that there were 76 genes with higher expression and 151 genes with lower expression in Class II compared with Class I. Among them, SPP1 was the most significantly differential gene with a LogFC of 2.36 (Figure 3B). We observed the expression and correlation of 120 traditional Chinese medicine targets in HCC (Figures 3C,D). VEGFA, an important target for HCC, was generally highly expressed in Class II (Figure 3C), so we hypothesized that Class II was more malignant than Class I. GSEA analysis showed that the gene set based on GO (Figure 3E) and KEGG (Figure 3F), Class II exhibits enhanced cell cycle and cell division processes, as well as reduced metabolic processes (especially P450 enzyme metabolism). The *p*-values of GSEA in this study were all less than 0.05, and the top5 items with higher NES were screened for presentation (Figures 3E,F; Supplementary table S4).This indicates that Class II is more malignant than Class I but less drug metabolic than Class I (Figure 3F **)**.

### Subtype characteristics and key genes of different subtypes of HCC

**FIGURE 4:**
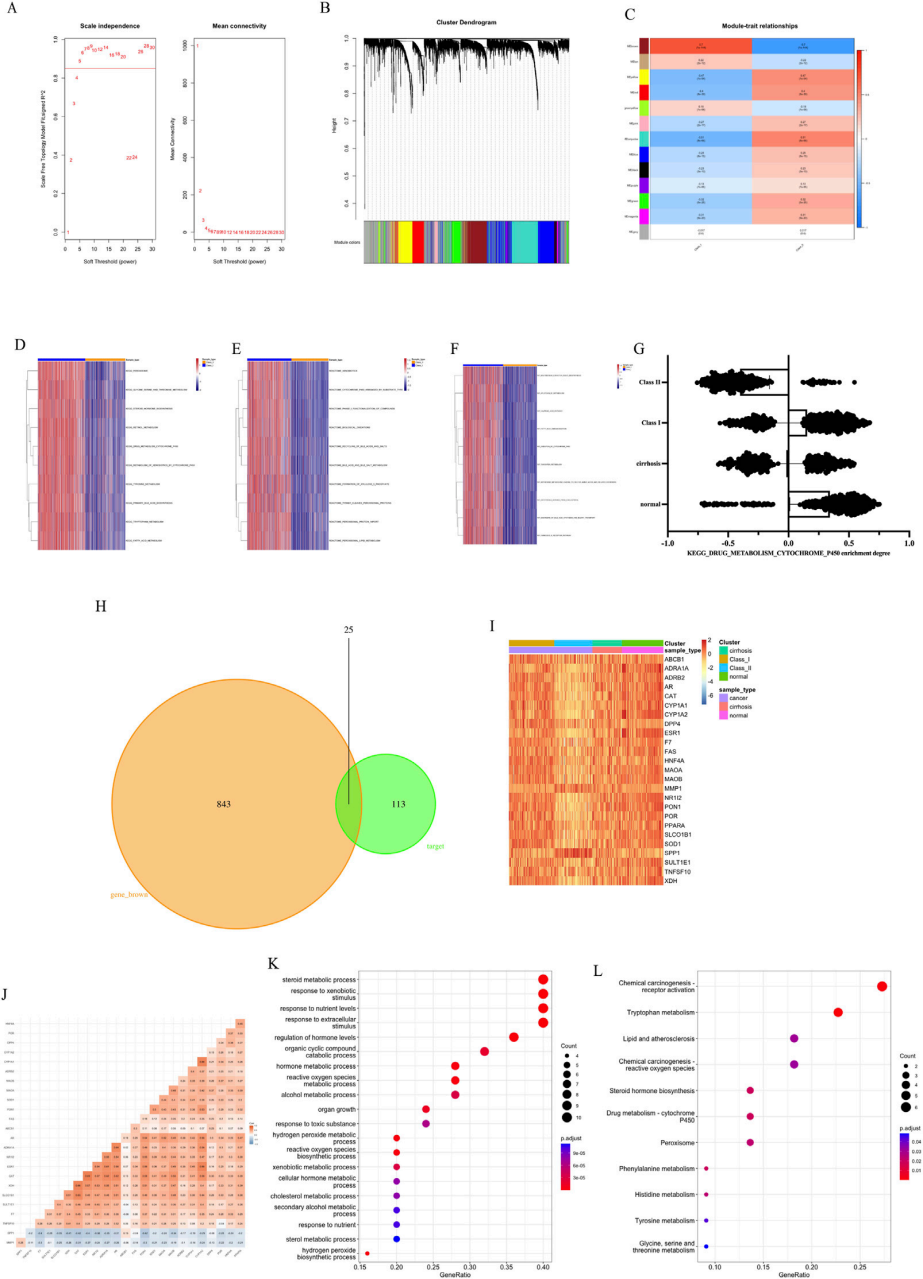
Subtype characteristics and key genes. **(A)** Soft threshold of WGCNA. **(B)** Cluster Dendrogram of WGCNA. **(C)** Module-trait relationships of WGCNA. **(D)** GSVA analysis by KEGG. **(E)** GSVA analysis by Reactome. **(F)** GSVA analysis by Wiki. **(G)** Metabolism of P450-related drugs by KEGG with GSVA analysis. **(H)** Intersection of MEbrown gene set and target genes. **(I)** Expression of 25 key genes. **(J)** Correlation of 25 key genes. **(K)** Gene enrichment Go analysis of key genes. **(L)** Gene enrichment KEGG analysis of key genes.

WGCNA analysis was performed on HCC sequencing data (Figures 4A–C). The soft threshold value obtained from the screening was 5 (Figure 4A). The analysis divided 9651 genes into 13 gene sets (Figure 4B). And the HCC subtype was found to be most correlated with the MEbrown gene set (r = 0.7, *p* < 0.05, Figure 4C). GSVA analysis was performed using three pathway gene sets, KEGG (Figure 4D), Reactome (Figure 4E) and Wiki (Figure 4F). The results of the study showed that there was a reduction in liver function-related metabolism in Class II compared to Class I, especially for P450-related drug metabolism. We also analyzed the metabolism of P450-related drugs in cirrhotic versus normal tissues by GSVA analysis (Figure 4G). The degree of Class II drug metabolism was found to be lower than cirrhosis versus normal tissue, but the degree of Class I drug metabolism was higher than cirrhosis and lower than normal tissue. This proximally confirms that Class II is less metabolizable and thus more malignant than Class I. The MEbrown gene set was intersected with the traditional Chinese medicine target set to obtain 25 key genes (Figure 4H). Among the 25 genes, Class II had low expression of 23 genes and high expression of 2 genes compared with Class I. SPP1 and MMP1 were more highly expressed in Class II and higher than in cirrhotic versus normal tissues (Figure 4I), and negatively correlated with the expression of other key genes (Figure 4J). Gene enrichment analysis revealed that these genes were associated with extracellular stimulation (Figure 4K) and with chemical carcinogenesis receptor activation (Figure 4L).

### Immune microenvironment characteristics and survival analysis of different subtypes of HCC

**FIGURE 5:**
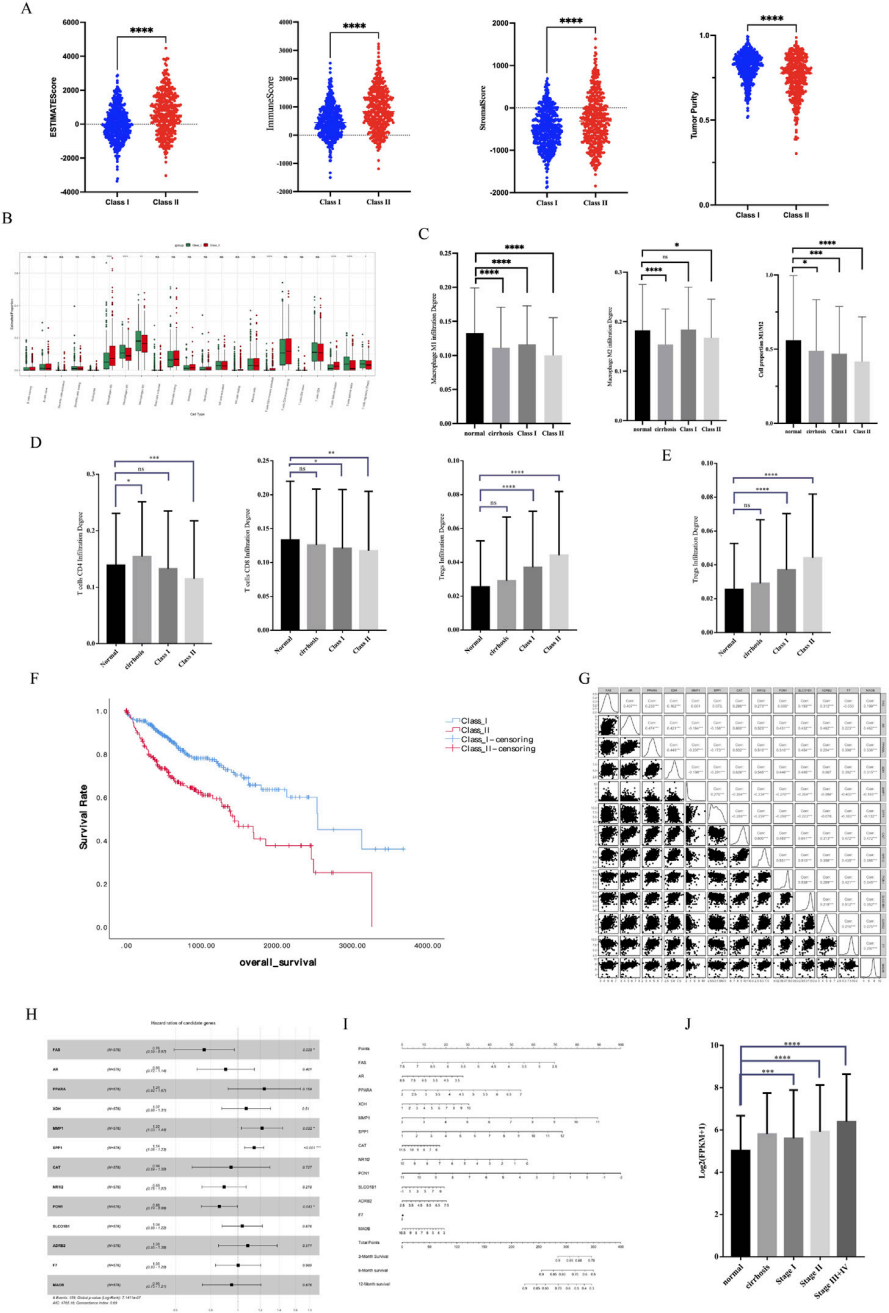
Immune microenvironment characteristics and survival analysis. **(A)** Estimate analysis of HCC. **(B)** Cibersort immune infiltration analysis. **(C)** Macrophage infiltration analysis. **(D)** CD4-positive and CD8-positive T-cell infiltration analysis. **(E)** Treg infiltration analysis. **(F)** KM analysis of different subtypes of HCC **(G)** Correlation of 13 key genes related to survival. **(H)** HR Forest Map of 13 key genes. **(I)** Nomogram of 13 key genes. **(J)** SPP1 expression in different stages of HCC.

Previous results demonstrated that Class II has a higher degree of malignancy and greater damage to liver function, as well as a lower metabolism. In this study, we first performed Estimate analysis of sequencing data from HCC patients (Figure 5A). We found that Class II had a higher Estimate score and had more immune cell components with stromal cell components. However, Class I had higher tumor purity than Class II. Cibersort immune infiltration analysis showed significant differences between Class I and Class II in terms of macrophage and T-cell infiltration (Figure 5B). In terms of macrophage infiltration (Figure 5C), there was a decrease in the M1 type ratio and an increase in the M2 type ratio in Class II, resulting in a lower M1/M2 in Class II. In terms of T-cell, there was a decrease in CD4-positive T-cell, a decrease in CD8-positive T-cell, and an increase in the CD4/CD8 ratio in Class II (Figure 5D). Of note is the presence of a higher infiltration of Treg cells in Class II (Figure 5E). In summary Class II has more extracellular matrix and suppresses anti-tumor immune responses in the microenvironment.

We performed a survival analysis of 576 HCC patients with clinical data. KM analysis showed that Class II had a worse prognosis and shorter survival time than Class I (Figure 5F). We constructed Cox survival regression models using 25 key gene expressions and screened 13 genes that were associated with survival time (Figure 5G). Among the 13 genes, four genes, SPP1, MMP1, PON1, and FAS, were significantly associated with survival (Figure 5H) and could predict the probability of patient survival (Figure 5I). In particular, it is important to note that SPP1, a key gene with significant differences, had high expression in advanced tumors (Figure 5J), suggesting that SPP1 may be an important oncogene in HCC.

### Multi-omics analysis of key genes and sensitive drug analysis

**FIGURE 6:**
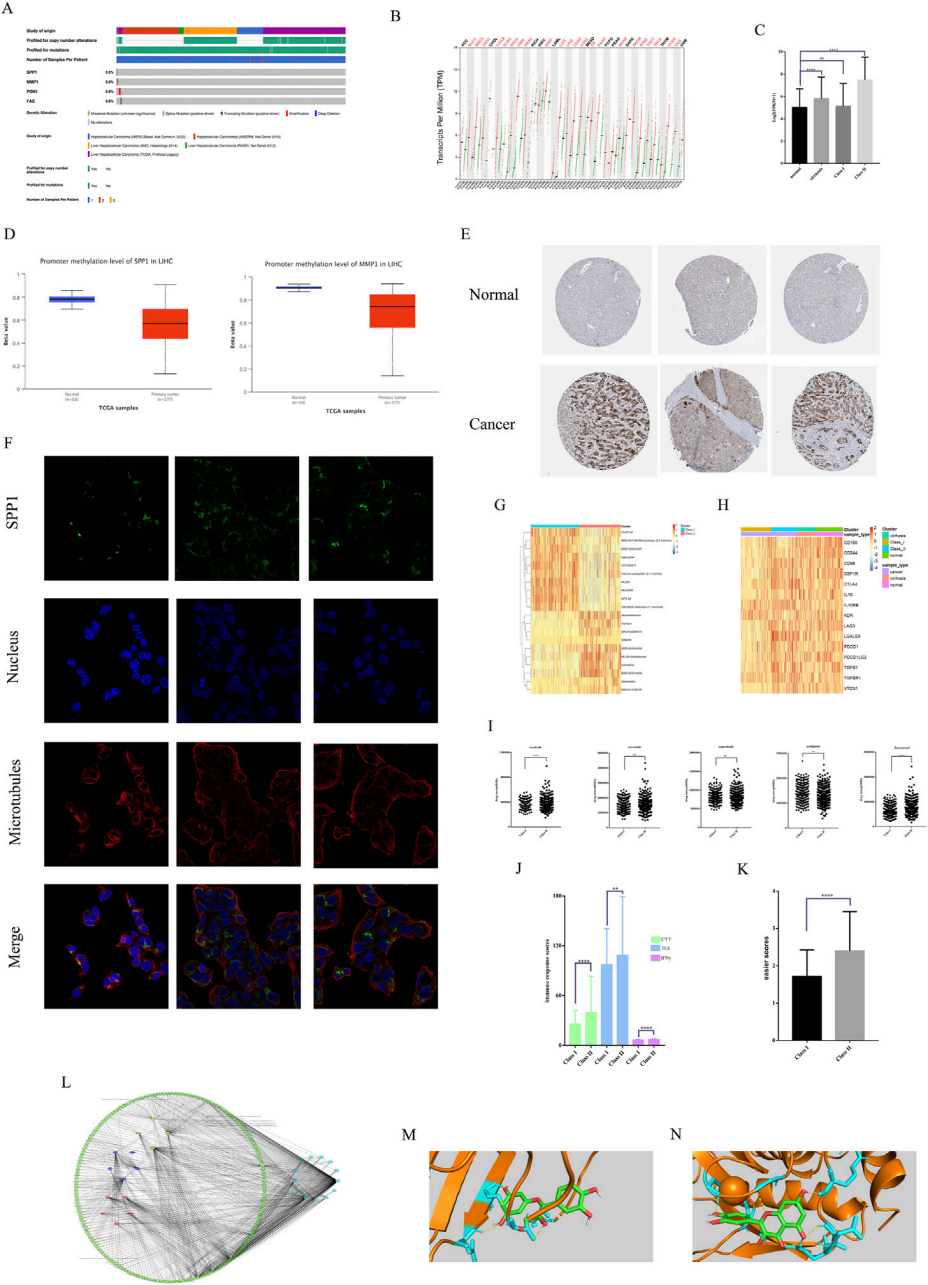
Multi-omics analysis and sensitive drug analysis. **(A)**Mutation analysis of key genes. **(B)** Pan-cancer expression of SPP1. **(C)** SPP1expression of different subtypes of HCC. **(D)** Gene methylation of SPP1 and MMP1 in HCC. **(E)** IHC analysis of SPP1 expression. **(F)** IF analysis of SPP1 subcellular localization. **(G)** The top 10 small molecule drugs sensitive in different subtypes. **(H)** ICIs expression in different subtypes. **(I)** Sensitivity analysis of commonly used HCC therapeutic drugs. **(J)** Three different immune response scores for different subtypes. **(K)** Easier scores for different subtypes. **(L)** Drug-component-target network for 13 key genes. **(M)** Molecular docking of SPP1 with quercetin. **(N)** Molecular docking of MMP1 with quercetin.

We perform further analysis of important key genes. Mutation analysis showed that SPP1, MMP1, PON1, and FAS all had low mutation rates in HCC, which demonstrated that all four genes were stably expressed genes (Figure 6A). Pan-cancer analysis showed that SPP1 was in high expression in most of the 32 tumors (Figure 6B). And the expression of SPP1 in Class II was significantly higher than that in Class I with normal tissues. And there was high expression of SPP1 in cirrhosis, which proximately suggested that SPP1 was associated with malignant phenotype (Figure 6C). Methylation analysis showed low methylation of both SPP1 and MMP1 in HCC (Figure 6D). IHC analysis showed high expression of SPP1 in hepatic tissues of HCC, but almost no expression in the mesenchyme (Figure 6E). IF analysis showed that HepG2 SPP1 protein was widely present in the cytoplasm and had a large overlap with microtubule regions overlap with the microtubule region, suggesting that SPP1 may have a greater relationship with HCC metastasis (Figure 6F).

We also analyzed the sensitivity of small molecule drugs with immunotherapy in HCC patients of two subtypes. The results showed that Class I had greater sensitivity to carboplatin (Figure 6G) versus oxaliplatin (Figure 6I), while Class II had greater sensitivity to sorafenib, fluorouracil (Figure 6I). We show the top 10 small molecule drugs sensitive to both subtypes **(** Figure 6G). Class II showed higher sensitivity to lenvatinib and regorafenib than class I, but there was no significant difference (Figure 6I). Class II had higher expression of immunosuppressive checkpoints than class I vs. normal tissue (Figure 6H). And easier package immunotherapy sensitivity analysis showed that Class II had high immune response scores (Figure 6J) and easier scores (Figure 6K). Therefore Class II and higher immunotherapy sensitivity than Class I. This suggests that Class II may be prioritized for targeted therapy and immunotherapy, and that herbal therapy may enhance the efficacy of both treatments. We screened 13 key genes related to survival for effective small molecules of traditional Chinese medicine (Figure 6L). Molecular docking validation showed that both SPP1 (Figure 6M) and MMP1 (Figure 6N) could interact with quercetin through hydrogen bonds with binding energies of −7.58 kj/mol and −7.22 kj/mol, respectively. This suggests that quercetin may be an important adjunctive therapeutic agent for HCC.

## Discussion

As the main type of liver cancer, HCC has a variety of treatment methods. At present, surgery is still the best treatment for HCC. But not all patients have the possibility of surgery, which requires other treatments to make patients have the opportunity for surgery. Chemotherapy and radiotherapy are traditional surgical adjuvant treatment methods, but the prognosis is poor and there are many adverse reactions. In recent years, with the development of genomics and tumor immunology, targeted therapy and immunotherapy have gradually replaced traditional therapy for adjuvant therapy and the treatment of advanced HCC patients. However, due to the single target of targeted therapy and immunotherapy, a large part of patients have no target, resulting in poor drug response and poor prognosis.

Traditional Chinese medicine is a national treasure of China and the main treatment method of traditional Chinese medicine. There are a variety of traditional Chinese medicines and prescriptions for the treatment of HCC. Relevant studies have shown that traditional Chinese medicine can affect the development, occurrence and spread of tumors and lead to imbalance within the tumor (Xi and Minuk, 2018). Traditional Chinese medicine can inhibit the invasion and metastasis of HCC, and is related to the EMT process of HCC (Jia et al., 2021; Li et al., 2021). At the same time, traditional Chinese medicine can promote tumor angiogenesis and the growth of tumor stem cells in HCC (Li, 2016; Wang et al., 2020b; Tang et al., 2020). The pharmaceutical preparations of traditional Chinese medicines are mostly extracts of traditional Chinese medicines, which have a variety of active ingredients and belong to different types of compounds. Among them, related studies have shown that alkaloids related to traditional Chinese medicine have anti-HCC effects and have certain development prospects (Liu et al., 2019a; Liu et al., 2019b). Most of the HCC-related components have immunomodulatory effects, which can promote the body’s immune response and facilitate the immunotherapy of HCC (Jia and Wang, 2020). This is consistent with the conclusion of this study. Traditional Chinese medicine injections commonly used in clinic are also used in combination with targeted therapy and immunotherapy.

Although there are many targets for the action of traditional Chinese medicine, most of them are still used as adjuvant therapy in clinic due to the complex composition and the slow mode of action of the drug. One study showed that taking it for more than 3 months in a row increased the 3-year survival rate of patients with HCC (Hou et al., 2022). Another study also came to similar conclusions that taking traditional Chinese medicine for more than 6 months increased the survival time of patients, and this was associated with multiple pathway enrichment (Zhang et al., 2022). However, traditional Chinese medicine still needs to be used in combination with a variety of therapies. Several studies have shown that the combined treatment of traditional Chinese medicine can improve the survival rate and quality of life of HCC patients compared with western medicine alone (Liao et al., 2015; Yang et al., 2017; Liu et al., 2019c; Liao et al., 2020).

However, traditional Chinese medicine cannot be used as the main treatment for HCC. This may be due to the fact that most of the traditional Chinese medicine preparations are oral and external medicines, which have a long action time and need to be taken for a long time. Compared with chemotherapy drugs, targeted drugs and immune preparations, traditional Chinese medicine lacks good preparations for intravenous infusion. Although the commonly used traditional Chinese medicine injection has a certain anti-cancer effect, it is prone to many adverse reactions due to its complex composition. This study explores the active ingredients of commonly used liver cancer injections in clinic, aiming to discover the main targets of traditional Chinese medicine in the treatment of HCC, which is conducive to the development of more comprehensive HCC-related traditional Chinese medicine injections, and provides ideas and preliminary ideas for the further development of traditional Chinese medicine anticancer drugs Research results.

## Data Availability

The original contributions presented in the study are included in the article/Supplementary Material, further inquiries can be directed to the corresponding author.
